# Translational ethics: an analytical framework of translational movements between theory and practice and a sketch of a comprehensive approach

**DOI:** 10.1186/1472-6939-15-71

**Published:** 2014-09-30

**Authors:** Kristine Bærøe

**Affiliations:** Department of Global Public Health and Primary Care, University of Bergen, Kalfarveien 31, Postboks 7804, NO-5020 Bergen, Norway

**Keywords:** Translation, Translational ethics, Theory-practice gap, Medical ethics, Bioethics, Self-reflexivity and ethical discipline

## Abstract

**Background:**

Translational research in medicine requires researchers to identify the steps to transfer basic scientific discoveries from laboratory benches to bedside decision-making, and eventually into clinical practice. On a parallel track, philosophical work in ethics has not been obliged to identify the steps to translate theoretical conclusions into adequate practice. The medical ethicist A. Cribb suggested some years ago that it is now time to debate ‘the business of translational’ in medical ethics. Despite the very interesting and useful perspective on the field of medical ethics launched by Cribb, the debate is still missing. In this paper, I take up Cribb’s invitation and discuss further analytic distinctions needed to base an ethics aiming to translate between theory and practice.

**Discussion:**

The analytic distinctions needed to base an ethics aiming to translate between theory and practice are identified as ‘movements of translation’. I explore briefly what would constitute success and limitations to these intended translational movements by addressing the challenges of the epistemological gap between philosophical and practical ethics. The categories of translational movements I suggest can serve as a starting point for a systematic, collective self-inspection and discussion of the merits and limitations of the various academic and practical activities that bioethicists are engaged in. I further propose that translational ethics could be considered as a new discipline of ethical work constructively structured around compositions of translational movements.

**Summary:**

Breaking the idea of translational ethics into distinct translational movements provide us with a nuanced set of conditions to explore and discuss the justification and limitations of various efforts carried out in the field of bioethics. In this sense, the proposed framework could be a useful vehicle for augmented collective, self-reflexivity among both philosophers and practitioners who are ‘doing bioethics’. Also, carefully designed, overall approaches combining justified, self-reflexive philosophical and practical efforts according to the suggested distinctions could be expected to realise – or at least improve a facilitation of – translation of ethics across the theory-practice gap.

## Background

Translational research in medicine requires researchers to identify the steps to transfer basic scientific discoveries from laboratory benches to bedside decision-making, and eventually into clinical practice. On a parallel track, philosophical work in ethics has not been obliged to identify the steps to translate theoretical conclusions into adequate practice. Some years ago, the medical ethicist A. Cribb suggested that it is now time to debate ‘the business of translational’ in medical ethics [1]. Although Cribb himself was not convinced that it would be ‘… useful to echo the wider call for translational medical research that moves from bench to bedside with a call for translational medical ethics that moves from argument to action’ , he argued for the usefulness of discussing translation to clarify the limitations and opportunities of the theoretical and practical achievements within the field of medical ethics.

In this paper, I take up Cribb’s invitation to debate the potential academic and practical achievements in the multi- and interdisciplinary field of medical ethics through the lens of translation. While welcoming this initiative as a golden opportunity for coordinated, collective scrutiny, debate and justification of the work carried out within this field, I argue that crucial analytical steps are called for to facilitate a self-reflective debate.

Firstly, it is necessary to differentiate between approaches that can be labelled as ‘translational ethics’ but are based on fundamentally different conditions. The theory-practice gap can refer to the particular gap between basic medical research and clinical implementation of effective interventions, i.e. the gap being bridged by translational medical research. Translational medical research requires researchers to identify the steps needed (1) to transfer basic scientific discoveries into useful health improving *interventions*, as well as (2) to ensure the *adequate implementation* of best clinical practice and healthcare decision-making [2]. The ethics related to bridging this particular gap between theory and practice in medicine encompasses all ethical issues encountered in translation from theoretical assumptions to developed medical interventions, i.e. ‘from bench to bedside’. Further distinctions can be made within this version of translational ethics by considering the involvement of different stakeholders and their responsibilities in the research, implementation and evaluation process.

This version of translational ethics can be understood as the *ethics of translational medical research*. This particular version of translational ethics will, for example, concern the use of animals for experimental or therapeutic purposes, requirements on patient consent at different phases of clinical trials, and the role of the pharmaceutical industry in directing innovative research. In the literature, there are many examples of this kind of ethical work (see e.g. [3–5]). Cribb, on the other hand, focuses on translational ethics in relation *to the internal gap between theory and practice within the field of ethics itself*. In the rest of the paper I reserve the label ‘translational ethics’ for intentional attempts to cross this particular gap within ethics. It is worth noting that this latter interpretation of translational ethics also encompasses basic concerns of ethics addressing the thematic particularities of translational medical research.

Secondly, it is necessary to identify and distinguish the various *translational movements* that can be carried out in ethics. These translational movements are not likely to bring about the complete translation of theoretical ethics into practice by themselves. However, as I will argue, these distinct translational movements can be combined into a coordinated process of ethical work structured according to distinct, successive phases (as in translational medical research) which translate theoretically justified, normative arguments into practice. In this respect, I endorse a more optimistic view than Cribb does on the possibility of translating between theory and practice in ethics.

Several presuppositions for this conceptually mapping of translational movements should be clarified from the start. I will assume that philosophical approaches are broadly understood as any normative-theoretical approaches to ethics independently of what discipline it emerges from. I will assume that such approaches differ fundamentally from practical approaches, in that the former have the mere aim of seeking ‘truthfulness’ in terms of rational justification, coherence, logical reasoning (consistency) and conceptual clarity, while the latter do not [6]. The truth-seeking aim of practical approaches is to find feasible, justifiable and practical solutions to the confronted ethical issues and act accordingly. In addition, practical approaches are *directly* connected to personal moral responsibility for one’s own actions, while theoretical approaches are not (although it might be argued that they should be considered *indirectly* responsible for consequences of what they recommend).

Further, I assume that since philosophical and practical approaches are carried out within fundamentally different conditions for reaching conclusions it gives us reasons to consider philosophical and practical conclusions as representing different categories of knowledge. Consequently, we should be aware of the distinction between the translations of knowledge that take place *within* one of the knowledge areas and translation that cuts *across* these two different areas. The specific structures of translation (or at least ‘structures of transference’) of knowledge from one context to another *within* an area of knowledge make for an interesting discussion in their own right, like the use of hypothetic, construed examples to justify theories and casuistic in real world, practical reasoning. Here, however, ‘translation’ involves the aim of bridging epistemologically distinct areas involving different kinds of training and competence.

Moreover, I also assume that the general structures describing the nature of translation in medical ethics also describe the general structures of translation in ‘doing ethics’ in general and bioethics in particular. Bioethics covers ethics related to any living entities, including the environment, and to related decision-making carried out at any level, i.e. clinical as well as political. Thus, the field of bioethics encompasses medical ethics, but also opens up a broader scope of ethical issues and stakeholders beyond medical ethics. I will refer to the general conceptions of translational ethics and translational bioethics to keep the scope of relevant issues as broadly defined as possible.

Finally, I do not claim that translational ethics would require a straightforward, top-down application of normative theory on clinical work, nor do I presume that the philosophers (broadly understood as theorists working on normative matters) have all the pertinent premises for a justified practice. Rather, I assume that the key to translational ethics can be found within complex structures of interplay between the two very different approaches of gaining theoretical and practical ethical knowledge, respectively. In this paper, I unpack this complexity by providing a topology of different translational movements in bioethics. This topology invites greater reflexivity among philosophers and practitioners working within the field of bioethics. Thus, it can also support better and more nuanced justifications of chosen translational approaches to bioethical issues on a case-to-case basis.

In the following section, I identify different approaches that promote translation by systematically reflecting on how the movement of translation can take place *across* different modes of ‘doing ethics’.

## Discussion

### Bridging the philosophical reflection-practice gap: In what direction? What kind of intervention? Whose responsibility?

There are two general directions in which to try and bridge the philosophical reflection-practice gap: from philosophical reflection to practice, or from practice to philosophical reflection. In the former, the object for translation is bioethical research carried out in academic institutions as theoretically justified reasoning and conclusions. In the latter, the object for translation is bioethics as it is carried out as practical reasoning and conclusions about what to do within the field of medicine or other bio-related activities (e.g. environmental interventions). Translation thus involves the transference of elements of knowledge production from one area to the other.

I will further analyse the movement of translation with respect to (a) the direction in which the translation occurs (from philosophical reflection to practice, or vice versa), (b) the nature of the intervention (i.e. academic work, political/moral engagement/involvement or implementation/adherence to particular methodological strategies for shaping practice) and (c) the locus of responsibility. I shall point out several categories of approaches that qualify as translational movements in bioethics, structured around these three distinguishable factors. I will first consider different versions of academic bioethics aiming for translation across the philosophical reflection-practice gap, before considering practical, bioethical approaches seeking to bring about translation across this gap.

### Translational philosophical bioethics

In this section, I will first discuss academic work that supports translation from philosophical reflections to practice, followed by academic work that initiates the translation of practical knowledge into philosophical reflection. In general, the responsibility for carrying out translational, *philosophical* bioethics must be on those being trained in *philosophical theory and reasoning*. This does not, as we will see, exclude competent, empirical research on on-going practice from playing a key role in this kind of translational work.

#### Translational philosophical bioethics research by researchers

The translation from theory to practice occurs when philosophical, bioethical research affects how bioethical practice is shaped. Cribb uses the term translation metaphorically to investigate whether translation applies as well in ethics as it does in biomedical research. He succinctly points out that theoretical, rigorous thinking cannot be turned into practical conclusions in a real-world context without losing its initial, distinct characteristics [1]. If we were to take translation at face value as an application of theoretical, normative conclusions in practice we would have to expect *equal outcome* in the conclusions reached by philosophical reasoning on the one side and practical reasoning on the matter on the other. This represents too strong and substantial a claim on what translation amounts to in an ethical setting since it presumes that either theoretical thinking will have to collapse into practical reasoning or practical reasoning will have to mirror theoretical reasoning in order for any translation to take place. Thus, we might have to reject the idea that any talk about bridging between theory and practice in ethics in terms of translation can be useful. Or, alternatively, we can make a normative definition of ‘translation’ that makes it meaningful to use the concept to capture the exchange that can be carried out across the theory-practice gap in ethics. My suggestion is to go for the latter alternative. I propose that ‘translation’ from philosophical normative reflection to practice refers to what is produced by philosophical work when it *facilitates practical conclusions on the terms of practice itself that can be theoretically justified*. This means that theoretical approaches do not need to presume the validity of one particular normative theory to settle practical issues, like for instance a specific utilitarian approach. Examples of translational, theoretical work would not have to include theoretically justified, substantial conclusions, such as the moral acceptability of enhancement interventions or genetic screening. The point here is that translation between the knowledge areas should avoid the presumption that theoretical approaches based on the characteristics of theoretical reasoning stands in any authoritative position to dictate the rightness of the practical conclusions arrived at on the conditions of a practice. Relevant examples on apt translational theory are instead methodological approaches to ethical education and deliberation that are developed with the aim of supporting and shaping political, professional or private practical conclusions while accepting pluralism with respect to the normative points of view of the stakeholders involved. Concrete examples of this kind of work are the non-conclusive framework of the four principles of biomedical ethics, the structured ‘Moral Case Deliberation’ approach and the framework ‘Accountability for Reasonableness’ developed to sustain fair limit setting in healthcare [7–9]. Carefully designed teaching approaches in health ethics are also relevant examples of this kind of work (see e.g. [10, 11]).

Taking these considerations into account, purely normative, theoretical research on *ideal premises* (i.e. simplified, limited premises that basically allow for discussion and justification of principles, not real world actions) cannot be characterised as being translational in its own right. In order to enable the success of this particular kind of translational movement, the philosophical work must address, as adequately as possible, the *relevant challenges of the real world of practice*. This means that the researcher must aim to base his or her normative approach as closely as possible on non-ideal features of human psychology and socio-empirical conditions. He or she must design the approach to counteract identified barriers to ideal decision-making, such as particular empirical features structuring the context (e.g. resource scarcity, geographic and demographic challenges), biased distribution of opportunities among individuals (e.g. to voice concern, deliberate, process information, reach a conclusion and act on it), and pragmatic barriers that undermine feasibility (e.g. organisation of information flow in institutions). In this respect, practice in terms of empirical psychological, social studies and pragmatic, organizational structures must inform philosophical reflection and justification of the underlying structures of normativity on a case-by-case basis. It is important to note that although drawing upon empiric information, these approaches do not lapse into ordinary practical reasoning. The aim is to facilitate normatively justifiable practical decision-making among stakeholders under certain constraints, not to arrive at practical conclusions about what to do as a policy or in particular contexts. If researchers engage in this latter activity and take a normative stand on what to do, they act as politicians or stakeholders. Their moral right to do so should be questioned as legitimate on a case-by-case basis.

The responsibility for identifying relevant premises, and for producing and coordinating academic work on ethics that *adequately*, and in a non-arbitrary manner, facilitates the translation of philosophically justified knowledge into practice, rests with theoretical researchers. The reason for this is that practitioners, per definition, do not approach ethical issues primary as philosophical issues.

#### Translation of philosophical work into bioethical practice by practitioners

The translation from philosophy to practice can also be carried out with the support of practitioners. It can be considered an ethical responsibility of people working within the field of medicine to seek well-justified solutions to the ethical dilemmas encountered in their practice. By seeking to identify and clarify such dilemmas and discuss solutions in light of theoretical perspectives, they may support the translation of philosophical theory into practice on philosophical premises. However, a lack of training in theory and philosophical reasoning may veil the recognition of an ethical dilemma in the first place. It may disturb focused deliberation and lead to invalid conclusions. In this respect, practitioners with a double competence in both practical and philosophical training (without requiring formal education) might be crucial for the success of this translational movement. Although the initiative of this form of translation rests on the practitioners, theoretically trained bioethicists can help to prepare easily understood presentations of normative theory to facilitate its success.

#### Translational meta-bioethics analysis

In addition to the translational philosophical bioethics research described above, another kind of translational philosophical work may focus on analytical discussions of the different conditions for translational bioethics. The approach presented in this paper is an example of this translational movement. This meta-theoretical perspective aims to have an *indirect* impact on real-world bioethical practice by suggesting distinctions and conditions to facilitate discussions on how to properly carry out translational bioethics. Again, the responsibility for adopting this meta-perspective must be on theoretical bioethics researchers given the theoretical nature of this investigation.

### Translational practice of bioethics

Bioethical *practice* encompasses all kinds of contextualised reasoning and practical conclusions, including policy-making, which shape the ethical formation of real-life, medical and environmental decisions and interventions. I will begin by accounting for practical activity that supports the translation *from* theory *into* practice, before I describe the practice that supports the translation *from* practice *into* theory. By locating the responsibilities of different actors involved in medical ethics, I will further distinguish the movements of translation in ethics.

#### Researchers’ translation of philosophical work into bioethical practice

Philosophers or theoretical researchers may take on the ethical task of shaping the field of practice themselves, according to principles and theories they find theoretically convincing. When researchers are involved in real-world, ethical decision-making *on practical terms*, the translation from philosophical reflections to practice can be realised in several ways. By ‘going political’ , they can lobby for the implementation of theoretically justified, bioethical policies. Philosophers such as Peter Singer and Thomas Pogge toil to translate abstract principles and theory into feasible policies, and advocate for these on practical, political terms [12, 13]. Brock has convincingly reported on the need to distort philosophical normative reasoning on the premises of non-philosophical argumentation in order to avoid unfortunate misunderstandings in practical policy-making [14]. In this respect, theoretically trained scholars may realise a translational movement by facilitating acceptance for a theoretically justifiable point of view. However, theoretical bioethicists who actively influence substantial policy-making with their own normative viewpoints should take precautions to avoid undue impact. Scholars have the responsibility of clarifying that one is then acting as a citizen and is merely representing the political opinion of one citizen – he or she is not due exclusive political authority as an expert on political questions.

At the same time, philosophers may indeed have a legitimate authoritative role to play as theoretically trained academics when teaching, as well as in political or ethical committee work. This applies when they are introducing and mentoring theoretically justified analytical approaches on practical terms, while aiming to structure processes of moral or political deliberation to promote understanding and to help others reach practical conclusions. In this way, they can bring about a version of translational movement through instructive guidance.

#### Researchers’ translation of practice into philosophical research

Researchers can also support translation from practice to philosophical reflection by engaging in practice themselves. This occurs when a researcher assumes the role of an action researcher and gets involved in ethical deliberation processes. Here they can support practitioners or other stakeholders in identifying and articulating ethical issues at stake and learn to speak ‘the same language’ as practitioners, i.e. express concerns and lines of reasoning in the way they do. An analysis of this knowledge-producing process can bring new ethical themes to the desk for philosophical reflection. The deliberative process may also reveal new data to the participating researcher that supports new meta-theories on the underlying structures of ethical perception and reasoning. This translation from practice to theory can then, in turn, be used in the translation from theory to practice in the translational, philosophical work described above.

Researchers can also support the translation from practice to philosophical reflection by ensuring that the philosophical issue they are discussing is not merely a theoretical construct but an accurate reflection of the issue as it presents itself to real-world stakeholders in non-idealised, practical terms. The stakeholders’ perceptions – as well as the discovery of previously unrecognised perspectives – may lead the researcher to reformulate the issue or even articulate previously overlooked dilemmas [15]. The researcher may test out the presentation of an issue before subjecting it to rigorous scrutiny, for example by arranging focus group interviews with stakeholders. In this way, philosophical research includes the translation of practice into the process of articulating the objective for normative, philosophical scrutiny. There is no guarantee that stakeholders’ perspectives are always relevant – they might, for example, be more focused on how to overcome minor practical challenges than the challenges involved in broader issues like organising a fair healthcare system. Nevertheless, the experiences and perspectives of real-world stakeholders should be recognised as potentially valuable sources to render philosophical research relevant.

#### Practitioners’ translation of practice into philosophical research

Practitioners can also realise the translation from practice to philosophical reflection. They can use channels to draw attention to pressing ethical issues that they experience in their work, e.g. by contacting media, or by approaching philosophical researchers directly. In this way, practical experiences can find their way onto the research agenda for theoretical scrutiny (and potentially back again to shape practice, if the prescribed strategies are implemented in the form of new policies). It is worth noting that there might be practical and cultural barriers (e.g. a lack of time or social resources and informal codes of loyalty) to seeking external guidance on ethical challenges experienced within the field of practice. In addition, involving a third party such as the media – who might be more interested in fronting spectacular cases than structural, everyday ethical challenges with no clearly identified wrongdoers – may skew the attention on concerns generated by this form of translational movement.

### Conditions for translational ethics

I have now identified distinct, translational structures within the field of bioethics. The list is not necessarily exhaustive, and I have merely hinted at what would constitute success and the limitations of the presented versions. I strongly believe that it should be considered a collective responsibility of the research community in bioethics to explore and debate the conditions for these different approaches as they promote a normatively justifiable, real world, bioethical practice. Debating the justified role that a bioethicist should play in various settings is a core task of a self-reflecting ethics research community. This task could benefit from more systematically organised attention. The same goes for the role of representatives of other disciplines within this field. Bioethics journals should strongly encourage and facilitate this type of self-reflecting work.

As mentioned earlier, the different translational movements should be considered to facilitate, rather than to actually realise, translation between theory and practice. For translation to be completed, it seems useful to adopt similar analytical phases that describe translational medical research: identification of the ethical challenge, development of a normatively justified approach, testing of the feasibility of the proposed approach in a real-world setting, implementation of the adjusted result, evaluation of the resulting practice, and if not successfully implemented (i.e. the ethical challenge still remains), the challenge of re-designing an adequate decision-making process or educational or mentoring effort is sent back for further scrutiny. The translational movements described earlier can be used in these various phases of bringing about translation; sets of self-reflexive, justified substantive conditions for translational movements can be coordinated for realising normatively valid translational ethics. The justified combination of distinct translational movements is likely to vary according to the kind of practice subject to ethical change (e.g. policy-making processes may call for other combinations of translational movements as compared to clinical challenges). The planning and designing of well-justified processes of translational ethics will thus be a crucial element of translational ethics itself. Translational ethics can now be described as an intended and purposive construction of theoretically justified, translational movements. ‘Translation’ in this sense is not taken at face value as theoretical reasoning being adopted by practice. Rather, the overall translational movement taking place between the epistemological different areas of theory and practice by this suggested overall strategy is better understood as a facilitation of well justified practices carefully respecting both theoretical and practical premises. Considered in this way, it occurs as a new academic discipline rather than, negatively defined, a compromise between theory and practice.

## Summary

In this paper, I have explored the conditions for translational ethics by identifying different categories of translational movements that address the theory-practice gap. As an analytical framework, the proposed overview of substantive versions of translational movements can serve as a starting point for a systematic discussion, and a self-reflective inspection of the knowledge-producing conditions of the multifaceted field of bioethics. As part of a coordinated approach, they can serve as building blocks in the overall process of constituting a new ethical discipline – translational ethics that cross the theory-practice gap within ethics.

## Author information

Kristine Bærøe is employed in a 50/50 position as an Associate Professor and a postdoc at the Department of Department of Global Public Health and Primary Care, University of Bergen. Her background is in philosophy. Her post doc project “Translational ethics: Bridging the theory-practice gap in medical ethics by translational approaches to core issues in medical research, clinical care, and public health” addresses the fundamental challenges of bridging the gap between theoretical and practical bioethics in a constructive manner.
